# Support-Enabled 3D Printing of Complex Anisotropic Hydrogel Structures Using Cellulose-Based Inks: Pathways Toward Biomimetic Human Aorta Models

**DOI:** 10.1021/acsbiomaterials.5c02127

**Published:** 2026-03-27

**Authors:** Daniel Pint, Tobias Steindorfer, Florian Lackner, Tamilselvan Mohan, Heidrun Gruber-Woelfler, Rupert Kargl, Karin Stana Kleinschek

**Affiliations:** † Institute of Chemistry and Technology of Biobased System (IBioSys), 685557 Graz University of Technology, Stremayrgasse 9, 8010 Graz, Austria; ‡ Institute of Process and Particle Engineering, 27253 Graz University of Technology, Inffeldgasse 13, 8010 Graz, Austria

**Keywords:** 3D bioprinting, direct ink writing, hydrogel, nanocellulose, sacrificial support, hydroxyethyl cellulose, alginate, cellulose bioinks, anisotropic printing

## Abstract

Three-dimensional (3D) printing of hydrogels has enabled new directions in biomedical engineering, soft material design, and biofabrication. However, unsupported hydrogel inks often collapse under their own weight or deform during printing, limiting achievable geometries. Herein, we present a reproducible, standardized support system combining a nanofibrillated cellulose/sodium alginate (NFC/ALG) structural ink with a cellulose-based sacrificial support ink composed of NFC, hydroxyethyl cellulose (HEC), and CaCl_2_. The process operates in open air, in contrast to immersion-bath methods such as FRESH, and uses a dissolvable support that locally stabilizes and initiates cross-linking of deposited filaments without submersion. Detailed protocols are provided for ink preparation, cartridge loading, direct ink writing (DIW) parameters, postprinting cross-linking, and support dissolution. Quantitative validation using tubular models shows that unsupported tubes collapse before 50 mm height, whereas supported tubes remain upright and stable. Surface fidelity is substantially improved by reducing layer height from 840 to 420 μm, yielding watertight prints. Demonstrations of complex geometries, including an anatomical aorta, DNA double helix, and the benchmark 3D Benchy model, confirm the method’s capability. Support dissolution in 30 mM CaCl_2_ occurs over approximately 3 days, ensuring stability during cross-linking and handling. Compared with other sacrificial strategies, this approach is inexpensive, cellulose-based, and relies on mild ionic cross-linking compatible with future cell-laden systems. This work provides a robust and accessible hydrogel engineering protocol, supporting stable and high-fidelity DIW of complex anisotropic structures.

## Graphical abstract

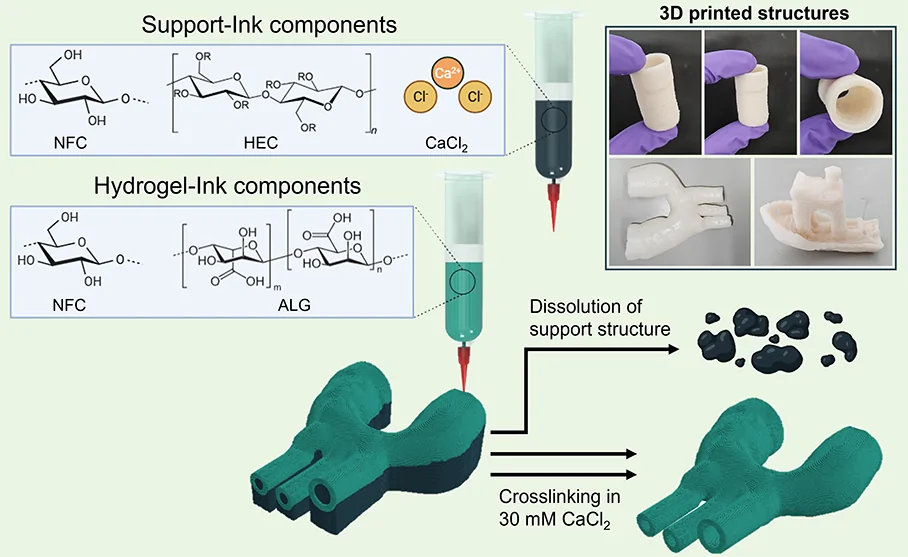

## Introduction

Extrusion-based three-dimensional (3D) printing of hydrogels has become a central technique in the fabrication of anisotropic, biomimetic scaffolds, soft devices, and engineered tissues.^1–3^ The process relies on hydrogel inks that exhibit shear-thinning and shear-force alignment, which allows extrusion through a nozzle, followed by rapid solid-like recovery to retain shape after deposition.^4–7^ Achieving this balance between flowability, structural stability, and anisotropy is critical to printing fidelity yet remains challenging for many hydrogel formulations.^3,8–11^

Most hydrogels require a cross-linking step to transition from their viscous state into a mechanically stable solid matrix. However, common cross-linking methods, such as photopolymerization or chemical cross-linking, can involve harsh conditions, slow kinetics, or exposure to reactive reagents that compromise biocompatibility and cell viability.^12,13^ Consequently, un-cross-linked hydrogel filaments often lack sufficient mechanical strength to support their own weight, leading to sagging, collapse, or tipping over of the structure.^14^ These mechanical limitations restrict the design of structures, hindering the fabrication of overhangs, hollow channels, or anisotropic high-aspect-ratio structures, features that are essential for applications such as vascular tissue engineering and bioprinting.^11,15–17^

To overcome these geometric and mechanical constraints, sacrificial or assistive printing materials including the FRESH printing method have been introduced to temporarily stabilize hydrogel constructs during deposition.^18,19^ Sacrificial inks can be coprinted with the structural material and later removed to act as support matrices that provide mechanical confinement during printing.^20^ However, many established systems rely on proprietary polymers, temperature-sensitive gels, or photopolymerizable networks, limiting their accessibility and reproducibility.^21,22^

Here, we report a reproducible cellulose-based sacrificial support ink designed to enhance the shape fidelity of nanofibrillated cellulose/sodium alginate (NFC/ALG) structural inks.^3,23,24^ The support formulation combines NFC (3 wt %), hydroxyethyl cellulose (HEC, 15 wt %), and CaCl_2_ (2.5 wt %), providing a shear-thinning and ionically cross-linking medium that stabilizes deposited filaments and enables the fabrication of complex, mechanically anisotropic 3D geometries.^11^ This cellulose-based support system is composed of biocompatible components widely used in biomedical and bioink formulations, low-cost, and environmentally sustainable.^25,26^ We demonstrate its utility in printing structurally stable tubular and branched architectures, illustrating its potential for advanced hydrogel constructs and vascularized tissue models.

## Materials and Methods

### Materials

Table 1 depicts the materials used in this protocol. Nanofibrillated cellulose (NFC, 3 wt % aqueous suspension, Sappi Valida S231C, 3%) was donated by Sappi (Maastricht, Netherlands) and characterized elsewhere.^27^ Alginic acid sodium salt (ALG, MW 300 000–350 000 g/mol, Carl Roth) and calcium chloride dihydrate (CaCl_2_·2H_2_O, ACS grade, Carl Roth) were purchased from Carl Roth (Karlsruhe, Germany). 2-Hydroxyethyl cellulose (HEC, average MW 250 000 g/mol) was purchased from Sigma-Aldrich (St. Louis, USA). Deionized water (ρ = 18.2 MΩ·cm) was used in all formulations.

**Table 1:** List of Chemicals and Materials Used^a^

Resource Chemicals	Source	Identifier
Nanofibrillated cellulose	Sappi, Netherlands	CAS: 9004-34-6
Calcium chloride dihydrate	Carl-Roth, Germany	CAS: 10035-04-8
2-Hydroxyethyl cellulose	Sigma-Aldrich, Germany	CAS: 9004-62-0
Alginic acid sodium salt	Carl-Roth, Germany	CAS: 9005-38-3

a The table summarizes all reagents employed for ink preparation, cross-linking, and printing, including supplier information and product identifiers. All chemicals were used as received without further purification unless otherwise specified.

### Equipment

- 50 mL Conical Centrifuge Tube (e.g., Nunc Conical Sterile Polypropylene Centrifuge Tubes 50 mL, Item no. 339652, Thermo Fisher Scientific, Waltham, MA)
- 24 mL disposable syringes (e.g., 24 mL HSW HENKE-JECT eco two-part disposable syringes, Item no. 8300063464, Henke Sass Wolf GmbH, Tuttlingen, Deutschland)
- Pneumatic extrusion cartridges (e.g., Optimum Syringe Barrels, Item no. 7366060, Nordson EFD, Westlake, Ohio)
- SmoothFlow Tapered Dispense Tips (840 μm inner diameter, Item no. 7018158, Nordson EFD, Westlake, Ohio)
- Polycarbonate plates (e.g., 4 mm RS Pro polycarbonate plastic plates, Gmünd, Austria)
- Agitator (e.g., IKA Eurostar 60 digital (30–2000 rpm), Ident-Nr. 0004446000, IKA-Werke GmbH & Co. KG, Staufen, Germany)
- Custom-designed high-speed stirrer (2000 rpm max) compatible with 50 mL Conical Centrifuge Tube (Figure S1, Supporting Information)
- Custom-designed syringe-cartridge connector (Figure S2, Supporting Information)
- Circular Shaker (e.g., Unimax 2010, Heidolph, Germany)
- GeSiM Robotics BioScaffolder 3.2, Version: 1.17.2.4575 (GeSim, Germany)
- Rheometer (modular compact rheometer MCR 502, Anton Paar, Austria)

### Methods

The experimental workflow was divided into six main steps, summarized below and in Figure 1, to provide an overview of the complete process:

**Figure 1:**
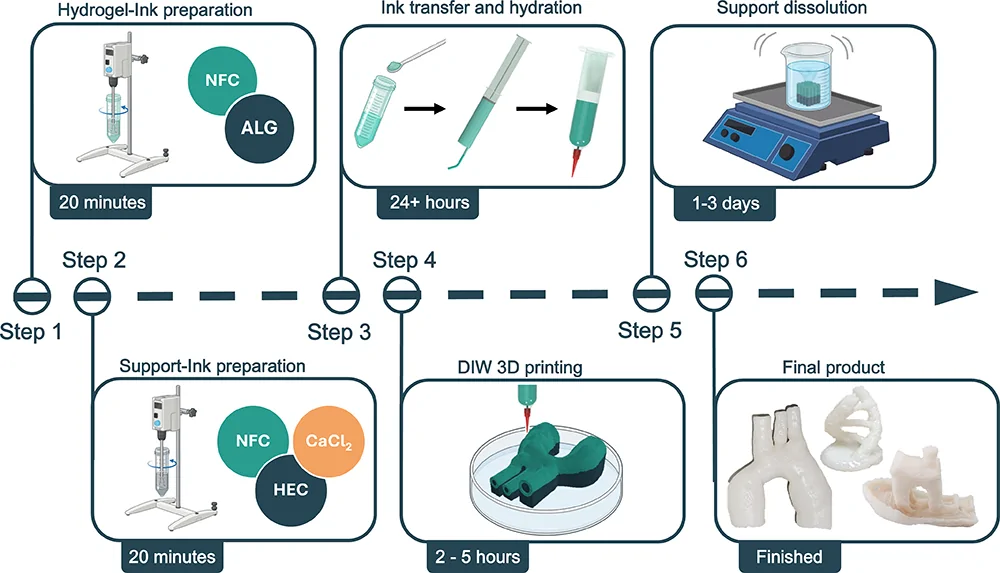
Schematic overview of the complete fabrication workflow. The process includes: (1) preparation of NFC/ALG structural ink, (2) preparation of NFC/HEC/CaCl_2_ sacrificial ink, (3) cartridge loading and printer setup, (4) direct ink writing of the complex geometries, (5) cross-linking in CaCl_2_ solution with simultaneous support dissolution, and (6) postprocessing to obtain freestanding hydrogel constructs.

**Step 1:** Preparation of structural ink (NFC/ALG)

**Step 2:** Preparation of sacrificial support ink (NFC/HEC/CaCl_2_)

**Step 3:** Cartridge loading and preparation

**Step 4:** Direct ink writing (DIW) 3D printing

**Step 5:** Cross-linking and support dissolution

**Step 6:** Postprocessing of printed constructs

#### Step 1: Preparation of Structural Ink (NFC/ALG)

- **Critical Parameters:** Mixing sequence, shear rate control, and agglomerate elimination
- **Time Required:** 45–60 min of active preparation
- **Storage Stability:** Up to 7 days at 4 °C in a sealed container

##### Material Preprocessing and Quality Assessment

- **NFC Suspension Evaluation:** Examine NFC suspension (3 wt %) for signs of settling, aggregation, or contamination. Acceptable suspensions exhibit uniform opacity without visible particles >1 mm. If settling is observed, gently invert containers 10–15 times before use.
- **ALG Powder Assessment:** Verify ALG powder (MW 300 000–350 000 g/mol) exhibits free-flowing characteristics without caking or discoloration (assess through appearance, properly stored ALG remains free-flowing).

The structural ink was designed to combine robust printability with postdeposition stability through a synergistic blend of nanofibrillated cellulose (NFC) and sodium alginate (ALG).^24^ NFC served as a rheological modifier and reinforcing component, imparting a pronounced shear-thinning behavior that enabled smooth extrusion through the nozzle and rapid viscosity recovery after deposition.^24^ Its entangled nanofibrillar network contributed to mechanical toughness, shape fidelity, and anisotropic reinforcement aligned along the printing direction.^3,28^

ALG was incorporated as an ionically cross-linkable polymer, complementing the NFC matrix by providing structural integrity and chemical tunability through Ca^2+^-mediated cross-linking. The addition of ALG increased the overall viscosity and yield stress of the ink while enabling controlled gelation during or after printing, thereby improving layer adhesion and dimensional accuracy. Together, these components yielded a composite hydrogel system capable of maintaining its printed geometry under gravitational and interfacial stresses during multilayer fabrication.

Formulations were prepared by dispersing ALG powder directly into the NFC suspension to achieve a final ALG concentration of 6.10 wt % in the total mixture; the exact amounts are shown in Table 2. To prevent agglomeration, ALG was premixed into the NFC suspension using a spatula prior to mechanical homogenization. Mixing was performed at 2000 rpm for approximately 5 min with a custom high-speed stirrer, while vertically agitating the tube to ensure uniform shear distribution. Ink homogeneity was verified visually under bright light by the absence of visible aggregates. Prepared inks were stored at 4 °C for up to 1 week and equilibrated to room temperature before printing to stabilize viscosity and extrusion behavior.

**Table 2:** Composition of the Structural Ink Formulations Used for Direct Ink Writing (DIW)^a^

Materials	Final Concentration [wt %]	Amount [g]
Nanofibrillated cellulose (NFC)	2.81	25
Alginic acid sodium salt (ALG)	6.10	1.625
Total		26.625

a Weighed amounts of each component and the resulting final concentrations (wt %) of nanofibrillated cellulose (NFC) and sodium alginate (ALG) in the final structural ink. Percentages are reported as weight fractions (w/w) based on the total mass of the prepared mixture. The NFC stock suspension contained 3 wt % solids.

For print visualization, 0.2 μL of red food dye was added to the NFC/ALG structural ink so that the structural and support phases could be readily distinguished. Prints prepared without a dye showed comparable outcomes.

#### Step 2: Preparation of Sacrificial Support Ink (NFC/HEC/CaCl_2_)

- **Time Required:** 60 min of active preparation + 24–48 h hydration
- **Storage Stability:** 7 days at 4 °C after complete hydration in a sealed container
- **Critical Success Factor:** Complete HEC hydration and homogeneous suspension determine final ink performance

The sacrificial support ink was developed to provide temporary mechanical reinforcement during the printing of soft hydrogel structures while remaining removable through controlled dissolution after fabrication. Building upon the NFC base formulation, the support ink was supplemented with hydroxyethyl cellulose (HEC) and calcium chloride (CaCl_2_) to tailor its rheological and interfacial properties for coprinting with the structural ink.

HEC acted as a water-soluble thickening and stabilizing polymer, enhancing the viscosity, thixotropy, and shape retention of the deposited filaments. Upon hydration, HEC established a reversible, entangled network that improved filament stability without permanent cross-linking, facilitating later removal of the support. CaCl_2_ was included as an ionic cross-linker to enable localized gelation of the alginate phase at the support–structure interface during coprinting. This transient cross-linking enhanced interfacial adhesion and construct stability, preventing collapse or delamination during layer-by-layer deposition.

The final formulation consisted of NFC (3 wt % suspension, corresponding to 2.49 wt % NFC solids in final formulation), HEC (15 wt %), and CaCl_2_ (2.5 wt %), shown in Table 3. To prepare the ink, the NFC suspension was transferred into a 50 mL centrifuge tube, followed by the addition of CaCl_2_, which was completely dissolved at 2000 rpm for 2 min. HEC powder was then gradually incorporated into the NFC/CaCl_2_ suspension and prewetted with a spatula to minimize clumping (Figure 2). Homogenization proceeded in staged mixing steps (1000 rpm for 2 min, 1500 rpm for 2 min, and 2000 rpm for 5–10 min) with intermittent manual agitation to ensure uniform dispersion. Mixing continued until no visible aggregates remained. The ink was stored at 4 °C for at least 24 h to allow full hydration of HEC, a prerequisite for consistent rheological performance. Before printing, the ink was equilibrated to room temperature to ensure stable extrusion and reproducible flow behavior.

**Table 3:** Composition of the Sacrificial Support Ink Formulations Used for Direct Ink Writing (DIW)^a^

Materials	Final Concentration [wt %]	Amount [g]
Nanofibrillated cellulose (NFC)	2.49	25
2-Hydroxyethyl cellulose (HEC)	15	4.518
Calcium chloride dihydrate (CaCl_2_)	2.5	0.798
Total		30.316

a The table lists the weighed amounts of each component and the resulting final concentrations (wt %) of nanofibrillated cellulose (NFC), hydroxyethyl cellulose (HEC), and calcium chloride (CaCl_2_) in the support ink. Percentages are reported as weight fractions (w/w) based on the total mass of the prepared mixture. The NFC stock suspension contained 3 wt % solids.

**Figure 2:**
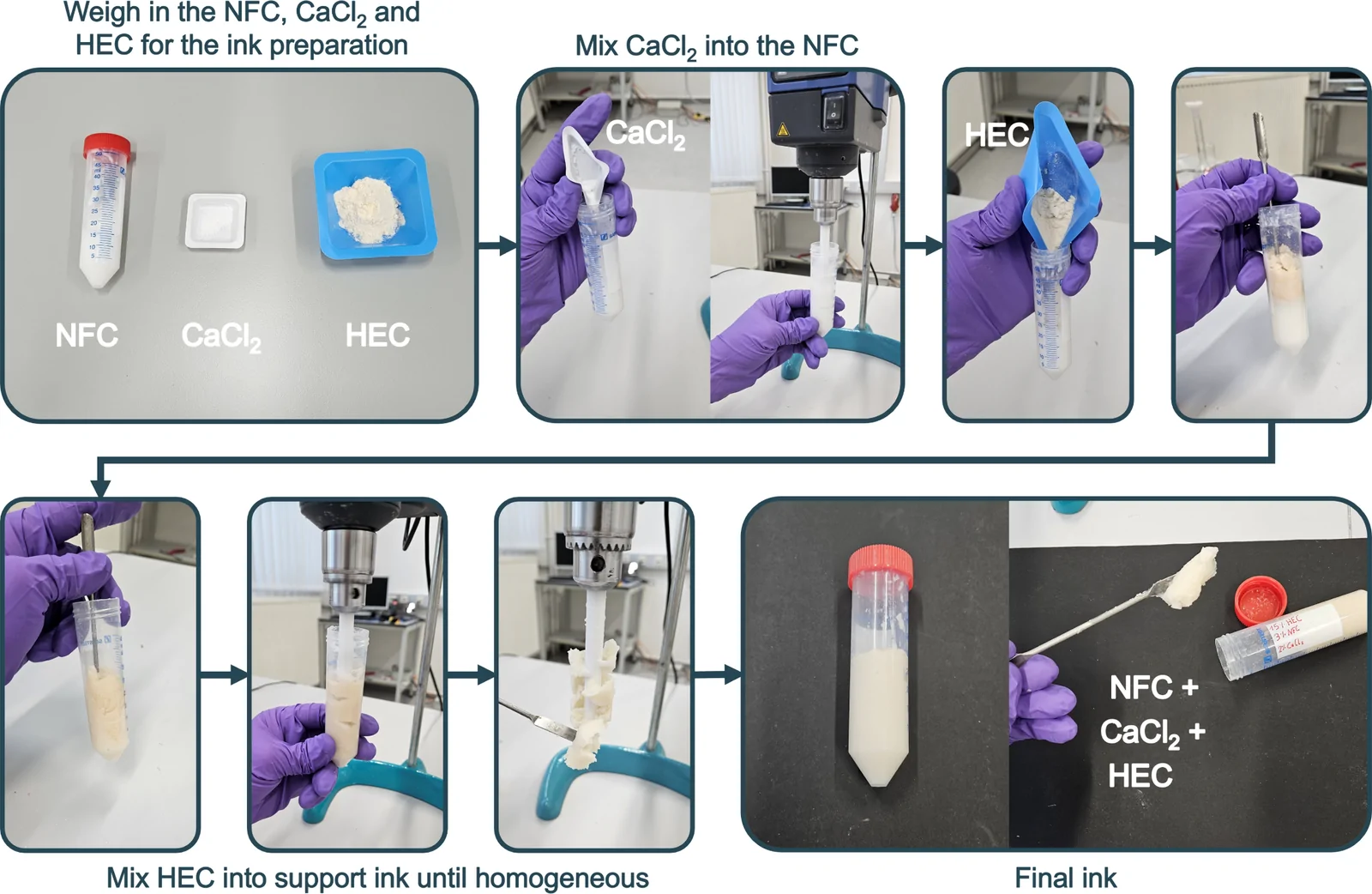
Flowchart illustrating the preparation of the sacrificial support ink. CaCl_2_ was first dissolved in the NFC dispersion, followed by the gradual incorporation of HEC under staged mixing. This process yielded a stable homogeneous sacrificial support ink suitable for direct ink writing.

##### Advanced Quality Control

- **Viscosity Assessment:** Properly hydrated ink should coat the spatula with a thick, even layer
- **Flow Behavior:** When tilted, ink should not flow and stay uniform
- **Color Uniformity:** Mixture should exhibit consistent opacity throughout without streaking and slightly yellow color
- **Rheological Measurements:** Rheological characterization of the structural (NFC/ALG) and support (NFC/HEC/CaCl_2_) inks were performed using rotational and oscillatory tests to evaluate viscosity, storage modulus (*G*′), and loss modulus (*G*″). All measurements were carried out on a rotational rheometer equipped with a parallel-plate geometry at 25 °C. Flow sweeps (viscosity vs shear rate) and amplitude sweeps (*G*′/*G*″ vs shear strain) were conducted to assess flow behavior and viscoelastic properties. A detailed description of the measurement parameters, instrument geometry, and rheometer settings is provided in the Supporting Information.

#### Step 3: Cartridge Loading and Preparation

- **Time Required:** 30–45 min for dual-ink setup
- **Critical Objective:** Achieve bubble-free loading with consistent ink distribution

The inks were transferred into disposable syringes using a spatula, and the material was carefully pressed against the syringe wall to minimize air entrapment, as depicted in Figure 3. Each syringe was filled to approximately 50% of its volume and gently compacted while keeping the syringe tip open to allow air to escape. The material was then transferred into pneumatic extrusion cartridges with a Custom-designed syringe-cartridge connector for subsequent use. The cartridges were preloaded with plungers filled to about 80% of their capacity to ensure uniform pressure transmission during printing. Depending on the intended timing of use, cartridges were either sealed to prevent drying out of the ink or immediately fitted with the appropriate nozzle for printing. All cartridges were labeled with the ink type, batch number, and preparation date to ensure traceability and reproducibility.

**Figure 3:**
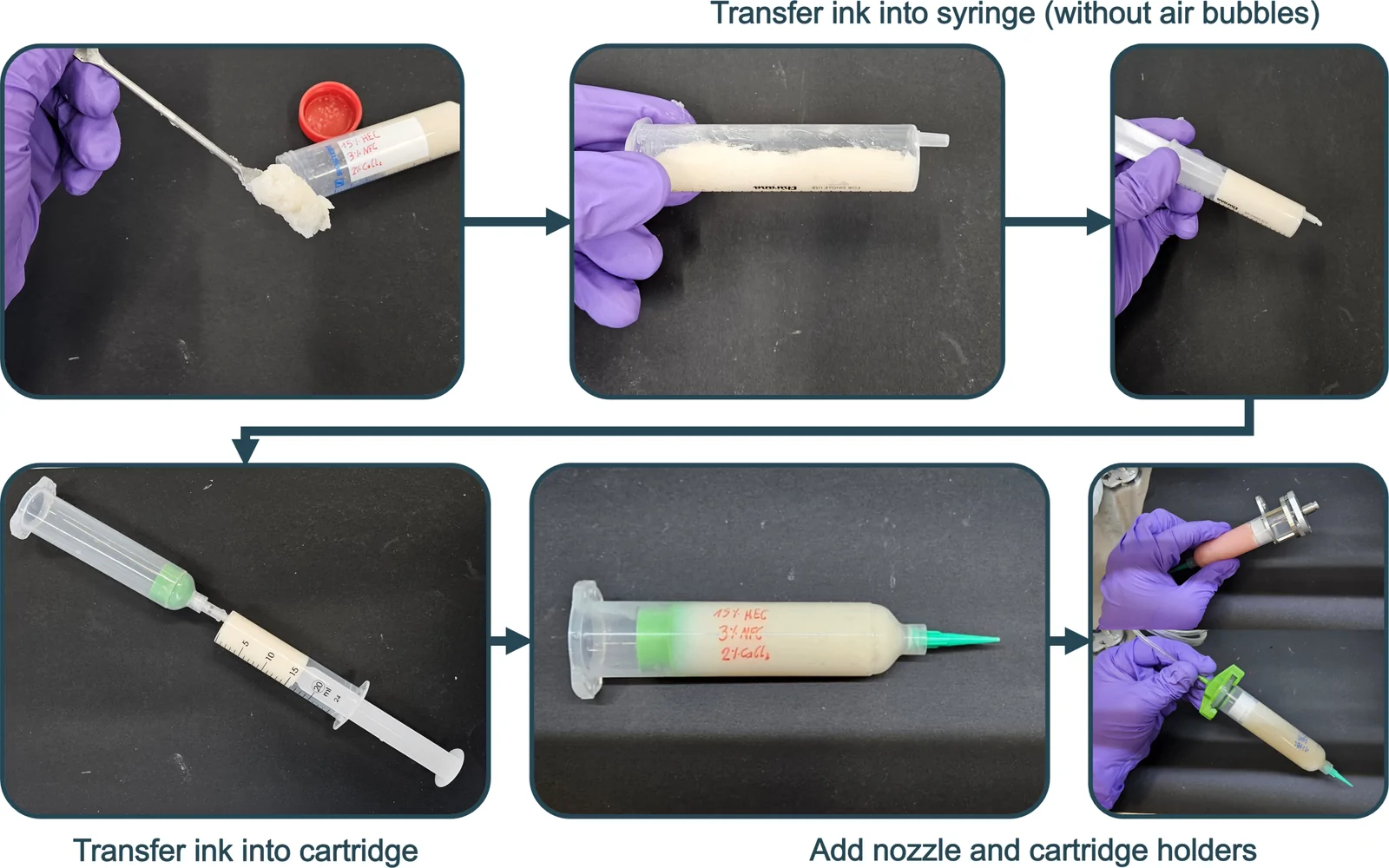
Flowchart depicting the transfer of the sacrificial support ink into cartridges. Following 24 h of hydration, ink was loaded into syringes, compacted to eliminate trapped air, and transferred into pneumatic extrusion cartridges. Cartridges were either equipped with extrusion nozzles if used immediately or sealed until used to avoid drying. Structural-ink cartridges were fitted with metal cartridge holders, whereas support-ink cartridges used in-house FDM 3D printed plastic holders to facilitate faster and easier cartridge switching.

##### Quality Control and Documentation

- **Cartridge Inspection:** Verify smooth plunger movement and absence of leaks
- **Extrusion Test:** Confirm consistent flow and absence of air bubbles by extruding a small amount of ink
- **Hydration:** Immediately seal nozzles with caps or tape to prevent ink drying out

#### Step 4: Direct Ink Writing (DIW) 3D Printing

- **Time Required:** 2–6 h depending on construct complexity
- **Critical Success Factor:** Correct setting of pressure, printing speed, and layer height

Printing was performed under ambient laboratory conditions (22 °C, 40–50% relative humidity) using an extrusion-based direct ink writing (DIW) system equipped with 580 or 840 μm inner-diameter nozzles. Prior to printing, the nozzle height was calibrated, and test lines were printed to establish the optimal extrusion pressure, print speed, and layer height for each ink. These three parameters were iteratively optimized based on strand uniformity, extrusion continuity, and layer adhesion, as illustrated in Figure 4.

**Figure 4:**
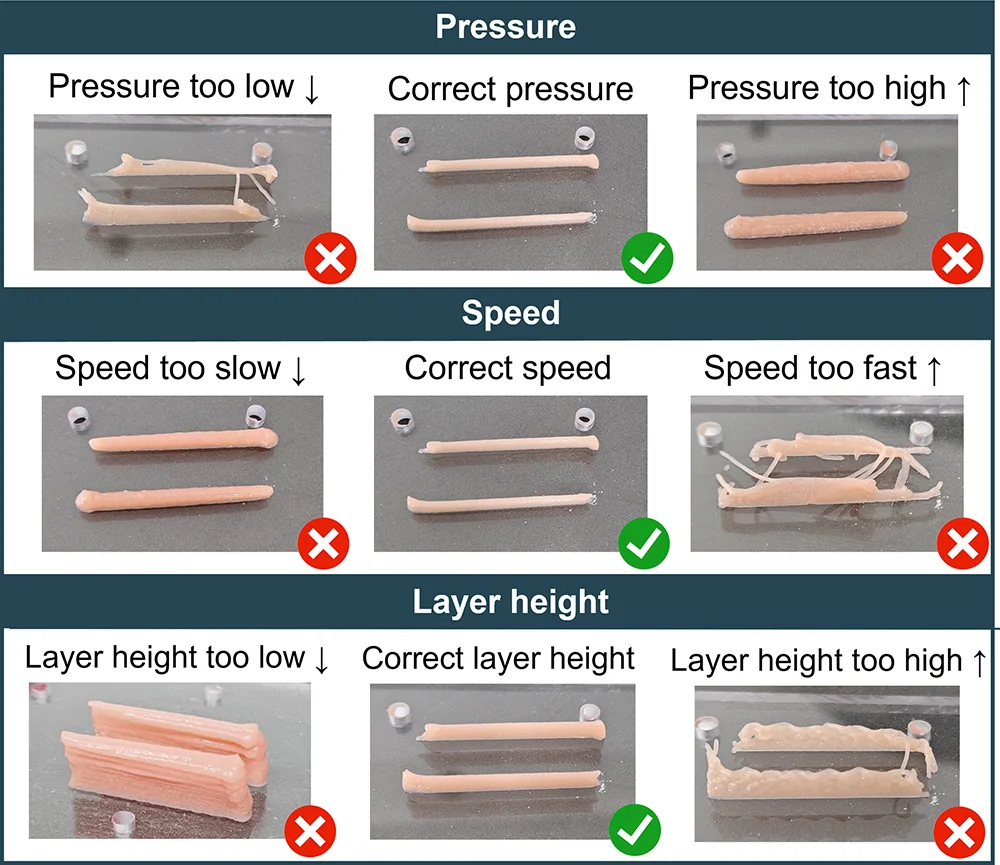
Optimization of the critical DIW parameters. Representative test lines (30 mm length) printed with the structural ink using a 580 μm nozzle illustrate the effect of extrusion pressure, print speed, and layer height on printing quality. Optimal parameters were selected based on strand continuity, width uniformity, and interlayer contact.

A layer height of approximately 50% of the nozzle inner diameter (e.g., 420 μm for an 840 μm nozzle) ensured sufficient ink compression for interlayer bonding without excessive deformation.^29^ During the first printed layers, extrusion pressure was tuned within the defined operational windows, 140–160 kPa for the structural ink and 175–235 kPa for the sacrificial support ink, to achieve smooth, continuous deposition and account for minor material or environmental variability. Layer height and print speed were identical for both inks. An initial speed of 10 mm s^–1^ was used for early test objects, and the final constructs were all printed at the targeted printing speed of 20 mm s^–1^, which balanced print time and quality. Different pressure settings were required for the two inks due to their rheological differences. All structures were fabricated using the same speed, layer height, and stated pressure ranges, with only support–structure spacing adjusted when needed. Typical printing durations ranged from 2 to 6 h, depending on construct complexity.

##### Advanced Printing Strategies

To further improve the construct stability, support structures were deposited in alternating orientations between layers, typically rotated by 90°, to enhance mechanical stability due to the anisotropic properties of the NFC, reinforcing the structure. This approach minimizes preferential failure directions and distributes mechanical stress evenly throughout the printed part.^3^ Support walls were maintained at a minimum thickness of approximately 2 mm to provide sufficient reinforcement without interfering with the later removal.

Interface management between the structural ink and the sacrificial support ink was systematically optimized to ensure structural ink stabilization during deposition while enabling selective removal of the support material after printing. The optimal interfacial clearance is not universal but depends on the nozzle inner diameter, line width, ink rheology, and geometric scale and complexity of the printed construct. For the nozzle sizes used in this study, a lateral separation distance of 0.2–0.3 mm provided reproducible structure-support contact without observable intermixing of the two inks.

Figure 5A shows the definition of the support clearance (offset) parameter in the GeSiM slicer interface used for toolpath generation (Further details are included in the Supporting Information Figure S3). The gap distance refers to the lateral clearance between the structural and support lines measured in plane, perpendicular to the printed path, from the centerline of the structural filament to the centerline of the adjacent support filament. The influence of the lateral gap distance on interfacial behavior is summarized schematically in Figure 5B. At insufficient spacing (0.0 mm), structural and support inks intermixed during deposition, compromising the interfacial definition and dimensional accuracy. At an optimal spacing of approximately 0.25 mm, stable filament support was achieved without mixing, enabling controlled interfacial ionic cross-linking and preserving surface quality. In contrast, excessive spacing (0.6 mm) resulted in intermittent contact between structural and support filaments, leading to filament sagging or local sinking and incomplete cross-linking in regions lacking sufficient support interaction.

**Figure 5:**
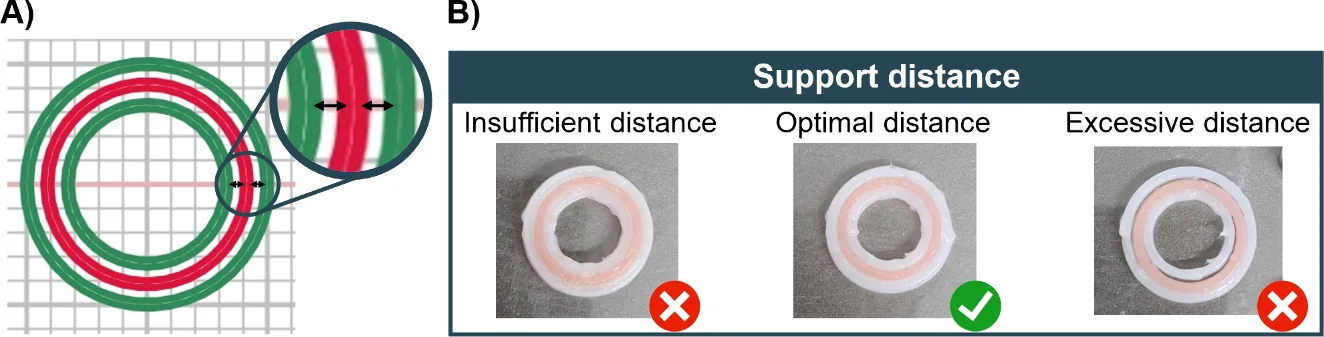
Definition and optimization of the lateral support clearance between structural and sacrificial support inks: (A) Screenshot of the GeSiM slicer interface illustrating the support clearance (offset) parameter used for toolpath generation. The gap distance is defined as the lateral in-plane center-line-to-center-line spacing between the structural filament and the adjacent support filament, measured perpendicular to the printing direction. (B) Schematic representation of three characteristic clearance regimes: insufficient spacing (0.0 mm), leading to intermixing of structural and support inks; optimal spacing (∼0.25 mm), enabling stable filament support without mixing and promoting localized interfacial cross-linking; and excessive spacing (0.6 mm), resulting in intermittent contact, potential filament sagging, and incomplete cross-linking in regions lacking support interaction.

As general guidance for comparable printing systems, an initial lateral clearance of approximately 50% of the nozzle inner diameter represents a rational starting point for subsequent optimization. This parameter should be refined empirically based on nozzle geometry, ink rheology, and structural complexity to ensure robust interface control.

#### Step 5: Cross-Linking and Support Dissolution

- **Time Required:** 3–4 days total (24 h active cross-linking + 2–3 days dissolution)
- **Critical Objective:** Achieve complete structural cross-linking while enabling controlled support removal

Immediately after printing, constructs were immersed in 30 mM CaCl_2_ solution for 24 h to cross-link the alginate component of the structural ink. During this period, partial softening and detachment of the support began. After 24 h, excess support material was gently removed manually and constructs were transferred into fresh CaCl_2_ solution on an orbital shaker (60 rpm). Complete dissolution of the sacrificial support occurred over approximately 3 days with 2–3 solution exchanges to accelerate removal. This staged process provided sufficient stability during cross-linking while ultimately enabling clean release of freestanding hydrogel constructs.

1. Immersion Protocol:

- **Volume Ratio:** Minimum 10:1 solution-to-construct volume for adequate ion availability
- **Container Selection:** Nonreactive materials (glass, polypropylene) with adequate depth
- **Positioning:** Ensure complete submersion without mechanical constraint

2. Quality Assessment Checkpoints:

- **2 h:** Verify structural ink maintains shape fidelity
- **6 h:** Confirm support softening without premature dissolution

3. Solution Exchange and Agitation Setup:

- **Fresh Solution:** Replace with new 30 mM CaCl_2_ solution
- **Agitation Parameters:** Orbital shaking at 60 rpm continuous operation
- **Container Considerations:** Ensure adequate clearance to prevent construct collision

#### Step 6: Post-Processing of Printed Constructs

After dissolution of the support, constructs were rinsed with deionized water and prepared for imaging or further analysis. Samples were photographed at each stage of fabrication, and representative geometries were subjected to evaluation of print quality, layer adhesion, and stability for validation of reproducibility.

## Results and Discussion

### Rheological Characterization

Rheological characterization was performed to evaluate the printability and mechanical behavior of both the structural NFC/alginate ink and the sacrificial support ink. Together, the flow curves, viscoelastic response, and yielding behavior define whether an ink can be smoothly extruded while maintaining shape fidelity after deposition. These rheological properties are essential for extrusion-based bioprinting and are well-defined criteria for hydrogel ink properties.^4,7,30,31^

### Shear-Thinning Behavior and Viscosity Hierarchy

Both formulations exhibited pronounced shear-thinning behavior with viscosity decreasing steadily with an increasing shear rate (Figure 6A). This behavior is characteristic of entangled polysaccharide networks such as nanofibrillated cellulose and hydroxyethyl cellulose, which align and disentangle under shear.^7,32^

**Figure 6:**
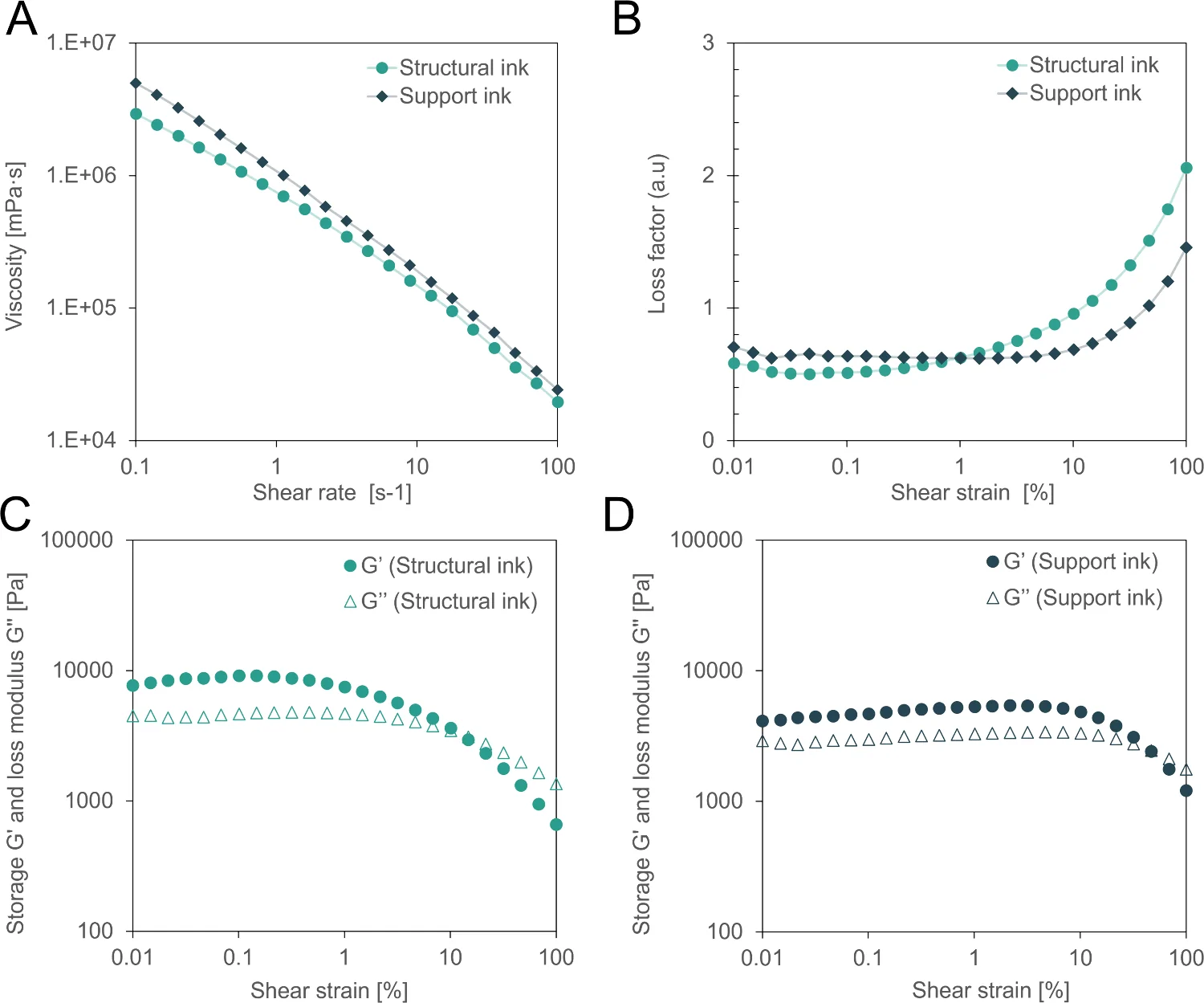
Rheological characterization of the structural NFC/alginate ink and the sacrificial support ink: (A) Viscosity as a function of shear rate, showing pronounced shear-thinning for both formulations and higher viscosities for the sacrificial support ink across all shear rates. (B) Loss factor (tan δ) vs shear strain. Both inks are initially solid-like with the sacrificial support ink exhibiting a slightly higher loss factor. The structural ink transitions to viscous behavior at ∼1% strain, whereas the sacrificial support ink remains elastic until ∼10% strain. (C) Storage (*G*′) and loss modulus (*G*″) of the structural ink with a rheological crossover at ∼10% strain. (D) Storage (*G*′) and loss modulus (*G*″) of the sacrificial support ink, yielding at ∼50% strain. These complementary behaviors support smooth extrusion of the structural ink and high deformation resistance of the sacrificial support ink during multimaterial printing.

Across the entire shear-rate range, the support ink displayed substantially higher viscosities than the structural ink, confirming its role as a mechanically more robust phase during multimaterial printing. A viscosity (η) hierarchy where η_support_ > η_structural_ is desirable because it ensures that the sacrificial support ink resists deformation while the structural ink can be extruded under moderate shear stress. Similar viscosity contrasts have been reported for support gels such as Pluronic F127 and gelatin-slurry supports.^18,20,30,33^ This hierarchy enables stable printing of overhangs, internal cavities, and high-aspect-ratio structures.

### Viscoelastic Properties and Loss Factor

Loss factor analysis (tan δ = *G*″/*G*′) was used to assess the balance between elastic and viscous behavior (Figure 6B). In the linear viscoelastic regime, both inks were predominantly solid-like (tan δ < 1). The sacrificial support ink showed a slightly higher loss factor (tan δ ≈ 0.7) than the structural ink (tan δ ≈ 0.6), indicating marginally greater viscous dissipation at very low strains. Nevertheless, both inks remained within ranges reported for printable polysaccharide gels.^7,32^

As strain increased, the structural ink transitioned into viscous-dominated behavior around ∼1% strain, while the sacrificial support ink maintained elastic dominance up to ∼10% strain. This difference in tan δ evolution demonstrates that the structural ink begins to flow under comparatively minor deformation, which is beneficial during extrusion, whereas the sacrificial support ink resists flow, contributing to its structural stability during printing.

Such strain-dependent viscoelastic separation between functional and sacrificial support inks has also been described for dual-ink GelMA/support systems and alginate-support bath formulations.^20,30^

### Yielding Behavior of Structural and Sacrificial Support Inks

Amplitude sweeps revealed distinct yielding behaviors for the two materials (Figure 6C,D). For the structural ink, the storage modulus *G*′ exceeded the loss modulus *G*″ at low strains, confirming solid-like gel behavior. The rheological crossover (*G*″ > *G*′) occurred at approximately 10% shear strain, indicating the onset of network breakdown and the transition into a flowing state. This relatively low yield strain supports smooth extrusion through fine nozzles, consistent with previous reports on NFC/ALG composite inks.^32,34^

The sacrificial support ink, in contrast, withstood substantially higher deformation before yielding. The crossover occurred only at ∼50% shear strain, demonstrating that the sacrificial support ink retains its internal structure over a much broader strain window. This behavior allows it to maintain mechanical stability during printing, act as a support scaffold for delicate features, and resist collapse during overhang formation. High yield strain has similarly been reported for HEC-based support materials and yield-stress support gels such as Carbopol and Pluronic systems.^18,20,30,33^

### Rheological Summary and Implications for Printing

Taken together, the rheological profiles of both formulations demonstrate a highly complementary behavior that is well suited for multimaterial extrusion printing. The structural NFC/alginate ink combines a comparatively stiff gel network at low strain with an early onset of yielding, enabling it to transition rapidly into a flowable state under nozzle shear, while still providing sufficient elastic recovery to maintain filament definition after deposition. In contrast, the sacrificial support ink exhibits a substantially higher viscosity across all shear rates, a delayed transition into viscous-dominated behavior, and a markedly higher yield strain. This combination endows the support material with pronounced resistance to deformation and allows it to preserve its structure under mechanical load, which is essential for stabilizing overhangs, internal cavities, and unsupported geometries during printing. Such complementary rheological characteristics mirror those reported for established dual-ink bioprinting systems and collectively provide the mechanical framework necessary for the reliable fabrication of complex, high-fidelity hydrogel architectures.

### Stability of Tubular Constructs with and without Support

To evaluate the stabilizing function of the sacrificial support ink, upright tubular models with fibers aligned along the radial direction were fabricated with an inner diameter of 12 mm and an outer diameter of 16 mm. Unsupported tubes printed solely from NFC/ALG structural ink exhibited progressive leaning above 40 mm height and collapsed below 50 mm (Figure 7A). Prior to failure, wall narrowing and asymmetric deformation were evident, consistent with compressive softening and buckling behavior typical of weak hydrogels under load. In contrast, tubes printed with the addition of one internal and two external NFC/HEC/CaCl_2_ support walls remained fully upright at 50 mm without visible deformation (Figure 7B).^28^ Supported constructs also withstood tilting of the Petri dish to 45°, retaining vertical alignment and demonstrating the capacity of the sacrificial support ink to distribute the mechanical load and prevent filament buckling.

**Figure 7:**
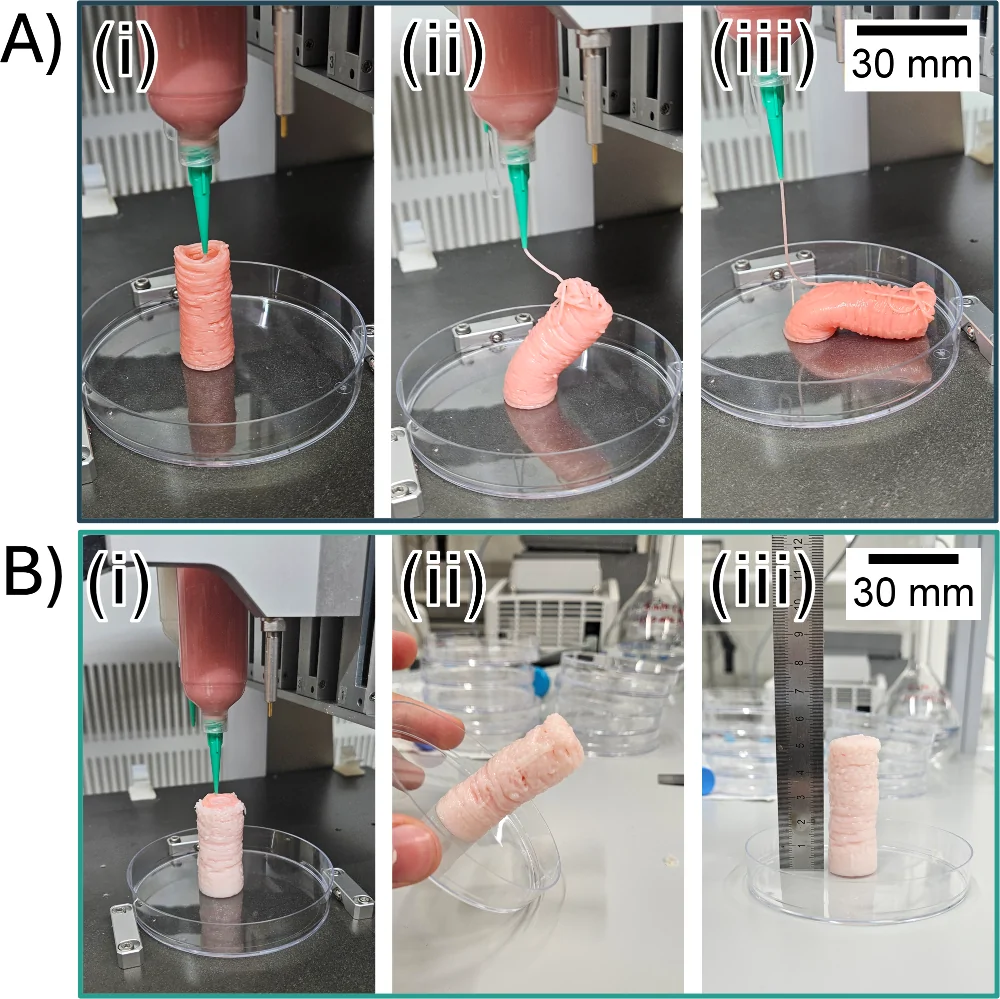
Comparison of unsupported and supported tubular hydrogel constructs: (A) Unsupported tube fabricated with NFC/ALG structural ink. (i) At 30 mm height, the construct remained upright but showed deformation at the upper edge. (ii) Progressive leaning and narrowing compromised the geometry. (iii) Collapse occurred below 50 mm height. (B) Tube printed with one internal and two external support walls of NFC/HEC/CaCl_2_ sacrificial ink. (i) White support layers are visible during printing at a 30 mm height. (ii) Stability was confirmed by tilting the Petri dish to 45°. (iii) The supported construct reached 50 mm height, remained upright, and showed no deformation prior to cross-linking.

These results align with prior observations that sacrificial or assistive materials are essential for high-aspect-ratio hydrogel constructs. Unlike immersion-based support strategies such as FRESH gelatin baths, the cellulose-based NFC/HEC ink forms self-standing supports and is directly compatible with open-air DIW printing, simplifying handling and reducing equipment requirements.^35^ The resulting 3D-printed anisotropic NFC/ALG materials have similar tensile strengths and elongations at break when compared to ex vivo porcine aortic tissue.^36^ Moreover, the open-air, self-supporting approach is not limited by immersion-bath depth and enables collision-free operation of the printhead, since no enclosing support bath constrains nozzle movement or print geometry. This combination allows unrestricted vertical and lateral fiber alignment and construct growth while maintaining high printing fidelity and spatial accuracy.^33,35^

### Influence of Layer Height on Surface Quality

Optimization of the layer height was critical to achieve smooth, sealed surfaces. At a layer height equal to the nozzle diameter (840 μm), tubular constructs displayed rough topography with deep grooves and visible pores between filaments (Figure 8A). Reducing the layer height to 420 μm, corresponding to 50% of the nozzle diameter, promoted partial spreading of deposited filaments onto the underlying layer, enhancing interlayer fusion and minimizing voids (Figure 8B).^29^ This adjustment resulted in watertight structures suitable for perfusion applications. While smaller nozzle diameters could theoretically improve resolution, such approaches are often limited by increased clogging risks in cellulose-containing inks. Thus, reducing the layer height while maintaining a larger nozzle diameter represents a practical compromise between fidelity and printing reliability.

**Figure 8:**
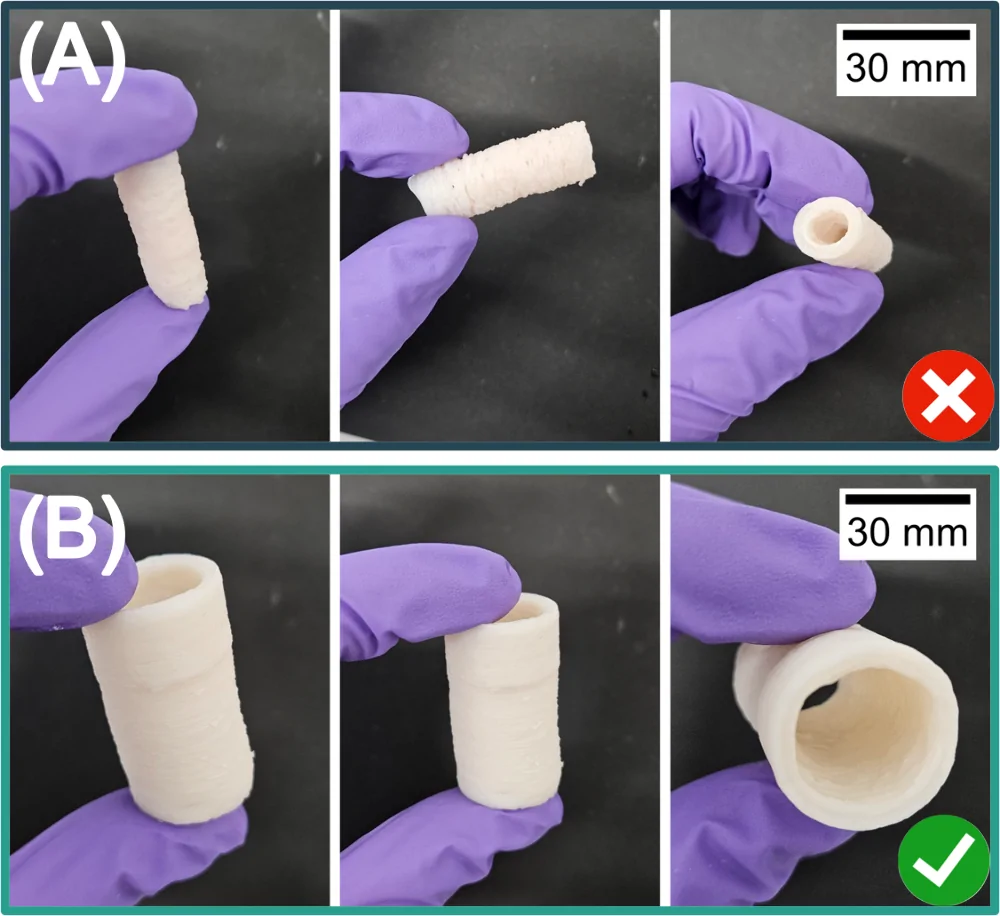
Effect of layer height on the surface quality of tubular hydrogel constructs. (A) Tube printed at a 840 μm layer height shows surface roughness, grooves, and visible pores. (B) Tube printed at a 420 μm layer height exhibits smooth, sealed surfaces with no visible pores, demonstrating improved fidelity.

### Printing of Complex Geometries

The method was further validated using four representative models designed to test different aspects of print fidelity: tubular structures (vertical stability), a human aorta (curvature and branching), a DNA double helix (continuous overhangs), and the benchmark “3D Benchy” boat (unsupported roofs and fine details), shown in Figure 9. Videos of a human aorta model printing are included in Videos S1 and S2.

**Figure 9:**
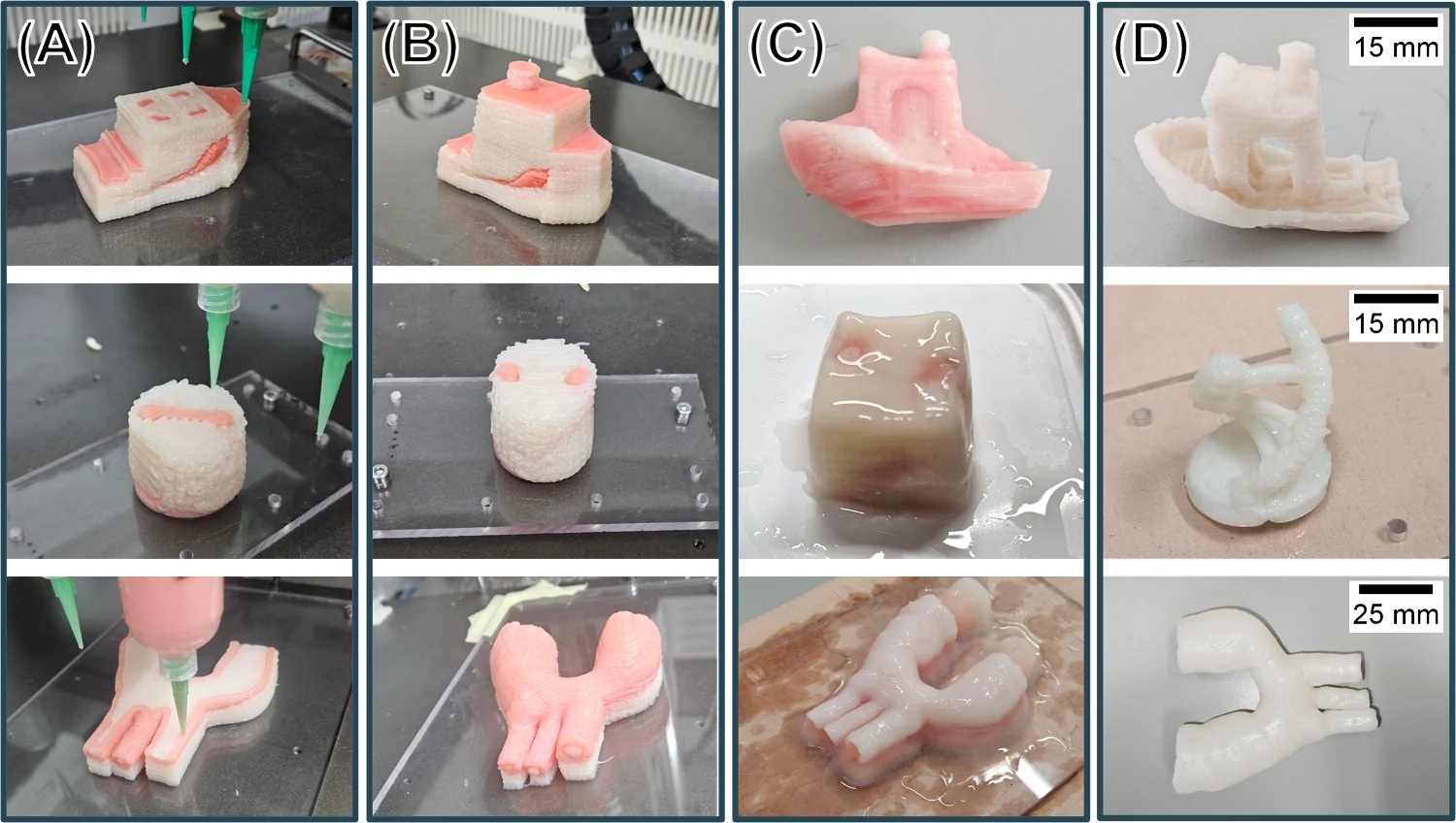
Validation of complex geometries fabricated with NFC/ALG structural ink and the NFC/HEC/CaCl_2_ sacrificial support. Representative models include the benchmark 3D benchy boat (top), DNA double helix (middle), and human aorta (bottom). (A) Constructs during midprinting (approximately 50% completion) with structural ink, tinted pink/red for visualization, deposited alongside white sacrificial support. (B) Immediately postprinting, showing full embedding of structures in the sacrificial support ink. (C) After 24 h in CaCl_2_ solution, partial cross-linking and initial support dissolution were evident. (D) After 3 days of cross-linking and dissolution, freestanding hydrogel constructs were obtained with preserved geometry and complete support removal. Note: the structural ink was temporarily tinted with food dye for visualization; final constructs are white after support removal and rinsing.

In all cases, constructs printed with sacrificial support retained geometry after cross-linking and complete dissolution. The DNA helix demonstrated uniform strand separation without structural collapse between turns, while the 3D Benchy model preserved delicate features, such as cabin walls and the chimney, with reduced sagging. The aorta model retained its curvature and lumen integrity after full support removal. These findings confirm that the NFC/HEC sacrificial support ink enables the replication of geometries that would otherwise be unprintable under open-air conditions.

### Sacrificial Support Dissolution Behavior

Following printing, constructs were cross-linked in 30 mM CaCl_2_ for 24 h to stabilize the NFC/ALG hydrogel network. During this period, the external support softened and could be manually removed, while internal sacrificial support ink dissolved gradually over 3 days under gentle shaking, shown in Figure 10. This extended time frame provided structural integrity during the critical cross-linking phase, while ultimately allowing full release of complex geometries.

**Figure 10:**
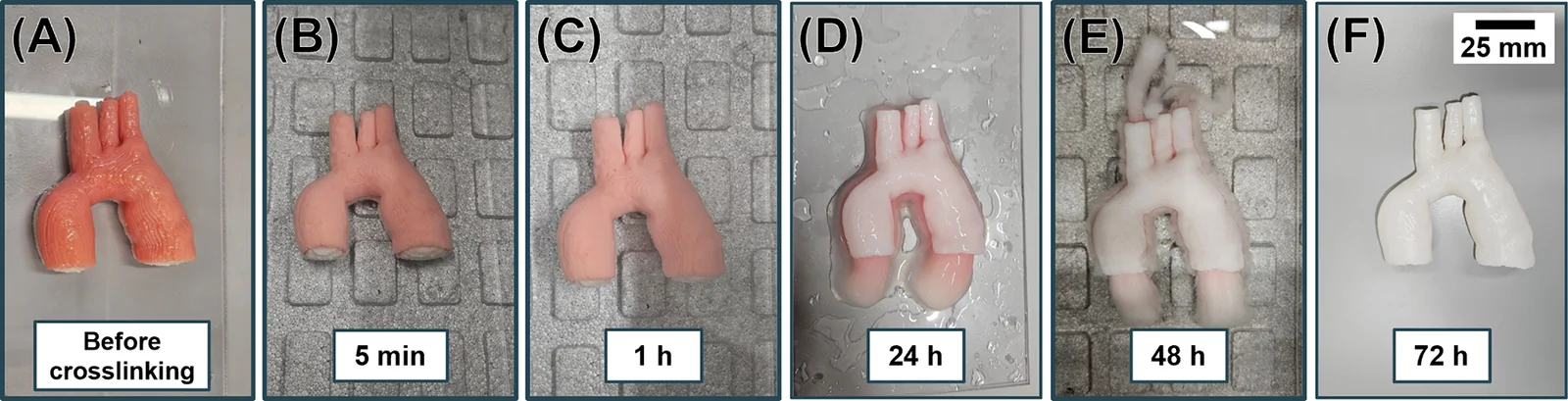
Time-lapse sequence of a printed hydrogel aorta during cross-linking and support removal in 30 mM CaCl_2_ solution. (A) Immediately after printing, the aorta retained its designed geometry with structural ink, tinted pink/red for visualization, and support (white) ink. (B) After 5 min of cross-linking, slight swelling was evident. (C) At 1 h, cross-linking progressed further. (D) After 24 h, the external support was largely dissolved, while the internal support detached gradually. (E) At 48 h, the internal support was expelled and partially removed manually. (F) After 3 days, complete dissolution of the support was achieved, yielding a stable freestanding hydrogel aorta. Note: the temporary tint was from food dye added for visualization; the final construct is white.

Although slower than those of gelatin-based sacrificial systems, the dissolution profile offers advantages by minimizing premature collapse. Potential avenues to accelerate dissolution include tailoring HEC molecular weight, adjusting Ca^2+^ concentration, or incorporating additives to enhance solubility. These strategies should be balanced against the need for adequate early stage stability.

### Troubleshooting and Process Reliability

Two recurring challenges were identified: nozzle clogging and layer height mismatch. Clogging occurred when ink mixing was insufficient, leaving agglomerated particles, or when nozzles were exposed to air, leading to drying at the tip. Both issues were mitigated by thorough premixing, extended homogenization, and sealing of the nozzles between prints. Layer height mismatches arose when extrusion pressures for the structural and support inks drifted apart, leading to uneven deposition. Real-time adjustment of pressures during printing resolved this issue, underscoring the importance of active process control in DIW workflows. A comprehensive guide for troubleshooting is given in Table 4.

**Table 4:** Troubleshooting Guide for DIW Support-Assisted 3D Printing

Problem	Symptoms	Root Cause	Immediate Solution	Prevention
Nozzle Clogging	Interrupted flow, pressure buildup	Inadequate mixing, dried material, contamination	Stop print, clear nozzle, adjust pressures	Thorough mixing validation, nozzle sealing, hydration
Layer Delamination	Poor interlayer adhesion, structural weakness	Incorrect layer height, printing speed	Adjust layer height to 50% nozzle diameter, adjust printing speed	Layer height optimization, printing speed optimization
Support Instability	Construct collapse, geometric distortion	Insufficient support density, poor support design	Increase support walls, modify support pattern	Support optimization protocol, adjust support design
Strand Inconsistency	Variable width, interrupted deposition	Pressure fluctuations, ink property variations	Real-time pressure adjustment, ink batch verification	Rheological validation, pressure system maintenance
Surface Defects	Rough texture, visible pores	Layer height mismatch, printing parameter issues	Re-examine printing parameters, surface postprocessing	Layer height validation, real-time quality control

### Comparison with Existing Approaches

Compared with existing sacrificial strategies, the NFC/HEC system offers several advantages. The materials are inexpensive, biocompatible, renewable, and readily available, in contrast to proprietary polymers such as Pluronic.^11^ The workflow requires no immersion bath, distinguishing it from gelatin-based supports, and relies on mild ionic cross-linking rather than UV or free-radical chemistry, which improves compatibility with potential future cell-laden applications.^25,37^

The main limitations are the slower support dissolution compared to gelatin-based systems and the resolution constraints imposed by the 840 μm nozzles. However, reducing layer height enabled watertight constructs without resorting to smaller, clog-prone nozzles. Taken together, the cellulose-based sacrificial support system represents a reproducible, accessible workflow for printing complex hydrogel geometries with high fidelity in standard DIW setups.

## Conclusion

In this work, we established a reproducible cellulose-based support strategy that significantly expands the capabilities of direct ink writing of hydrogel structures. By combining a nanofibrillated cellulose/sodium alginate (NFC/ALG) structural ink with a sacrificial support ink composed of NFC, hydroxyethyl cellulose (HEC), and CaCl_2_, we demonstrated that complex and mechanically fragile geometries can be fabricated with high fidelity. Tubular constructs that collapsed below 50 mm when printed unsupported were stabilized to the same height with supports while maintaining constant diameter and upright orientation. Furthermore, reducing the layer height from 840 to 420 μm transformed prints from porous and rough to watertight and sealed, providing surface qualities suitable for fluidic models and biomedical applications.

The method’s robustness was further validated through the successful fabrication of complex benchmark geometries, including an anatomical aorta model, DNA double helix, and the benchmark 3D Benchy. These models illustrate the versatility of the approach, ranging from bioinspired conduits with anisotropic biomechanics to intricate overhangs and fine structural details. In addition, the mechanical response of NFC/ALG constructs can be tuned via print-path design: altering the printing direction of the ink (e.g., circumferential, longitudinal, or lateral) modulates printing-induced anisotropy and enables adjustment toward aortic-like behavior, consistent with prior observations in similar NFC/ALG systems.^3,11^ Support dissolution in 30 mM CaCl_2_ required approximately 3 days, a trade-off that ensures structural integrity during cross-linking and handling but may limit throughput for rapid prototyping.

Compared with existing sacrificial support strategies, the NFC/HEC system provides several key advantages: (i) it is based entirely on inexpensive, renewable, biodegradable, and widely accessible cellulose derivatives; (ii) it enables open-air printing and does not require immersion support baths or specialized equipment such as temperature-controlled tanks such as those used in FRESH printing and can be implemented on open-source printing systems;^38^ (iii) it leverages mild ionic cross-linking rather than UV or radical-based curing, reducing potential cytotoxicity; and (iv) it is straightforward to reproduce across operators as tested in our laboratory. These characteristics address current challenges in accessibility and reproducibility that have limited the broader adoption of sacrificial support techniques.

The main limitations are the relatively slow dissolution kinetics and the reliance on large nozzles for reliable extrusion. Nevertheless, the method can be readily refined through molecular tailoring of HEC to accelerate solubility, optimization of CaCl_2_ concentration, and adaptation to finer nozzles. Importantly, the ionic cross-linking chemistry and mild processing conditions make the protocol inherently compatible with cell-laden bioprinting, as already validated in our previous study,^37^ opening future avenues in tissue engineering, regenerative medicine, biohybrid devices, and engineered living materials (ELMs).

In summary, this protocol contributes to a sustainable, open-source method for stabilizing hydrogel prints during DIW. By bridging the gap between accessibility and fidelity, it provides both a practical tool for hydrogel engineers and a platform for further development toward cell-laden systems and advanced biomedical applications.

## Supplementary Material







## Notes

Figure S1: Custom-designed high-speed stirrer used for ink preparation; Figure S2: Custom-designed syringe–cartridge connector for ink transfer; Figure S3: Digital workflow and automatic support generation; Detailed information about rheology measurements; Detailed information about design, support generation, and slicing workflow (PDF)
Video S1: Timelapse of 3D printing of a hydrogel aorta structure using the GeSiM Robotics BioScaffolder 3.2. (MP4)
Video S2: Timelapse of 3D printing of a hydrogel aorta structure using the GeSiM Robotics BioScaffolder 3.2. from a different angle (MP4)
