# Supplementary material for: Support-Enabled 3D Printing of Complex Anisotropic Hydrogel Structures Using Cellulose-Based Inks: Pathways Toward Biomimetic Human Aorta Models
Source: ACS Biomater Sci Eng. 2026 Mar 27;12(7):3319–29. doi: 10.1021/acsbiomaterials.5c02127 (PMC13637020; doi:10.1021/acsbiomaterials.5c02127)
Supplement: Supplementary file 1 [file ab5c02127_si_001.pdf]

Supplementary information

Support-Enabled 3D Printing of Complex Anisotropic Hydrogel Structures Using Cellulose-Based Inks: Pathways Toward Biomimetic Human Aorta Models

Daniel Pint,[a] *Tobias Steindorfer,*[a] *Florian Lackner,*[a] *Tamilselvan Mohan,*[a] *Heidrun Gruber-Woelfler,*[b] *Rupert Kargl,*[a,*] *and Karin Stana Kleinschek*[a]

*corresponding author

[a]Institute of Chemistry and Technology of Biobased System (IBioSys) Graz University of Technology, Stremayrgasse 9, 8010 Graz

[b]Institute of Process and Particle Engineering, Graz University of Technology, Inffeldgasse 13, 8010 Graz, Austria

E-mail: pint@tugraz.at, rupert.kargl@tugraz.at

Number of Pages: 7 (Pages S1-S7)

Number of Figures: 3 (Figures S1-S3)

Number of Videos: 2 (Videos S1-S2)

Abbreviations

3D	Three-dimensional
ALG	Alginic acid sodium salt (sodium alginate)
$CaCl_2$	Calcium chloride
DIW	Direct ink writing
G′	Storage modulus
G″	Loss modulus
HEC	Hydroxyethyl cellulose
MW	Molecular weight
NFC	Nanofibrillated cellulose
NFC/ALG	Nanofibrillated cellulose/sodium alginate structural ink
NFC/HEC/$CaCl_2$	Nanofibrillated cellulose/hydroxyethyl cellulose/calcium chloride support ink
PETG	Polyethylene terephthalate glycolate
tan δ	Loss factor

1. Ink preparation

1.1 3D printing of custom designed high-speed stirrer

To minimize dead zones during mixing in a 50 mL centrifuge tube, a custom stirrer was designed using the open-source software FreeCAD 0.19. The model was fabricated on an ANYCUBIC Photon Mono 4K resin printer (ANYCUBIC, China) using a UV-curable ABS-like resin. The propeller-type stirrer can be mounted onto a standard mechanical laboratory stirrer (e.g., IKA Eurostar 60 digital) and operated at 2000 rpm without mechanical failure. Tensile testing of printed specimens yielded a tensile modulus of 0.84 ± 0.07 GPa and tensile strength at break of 46 ± 2 MPa.[1]

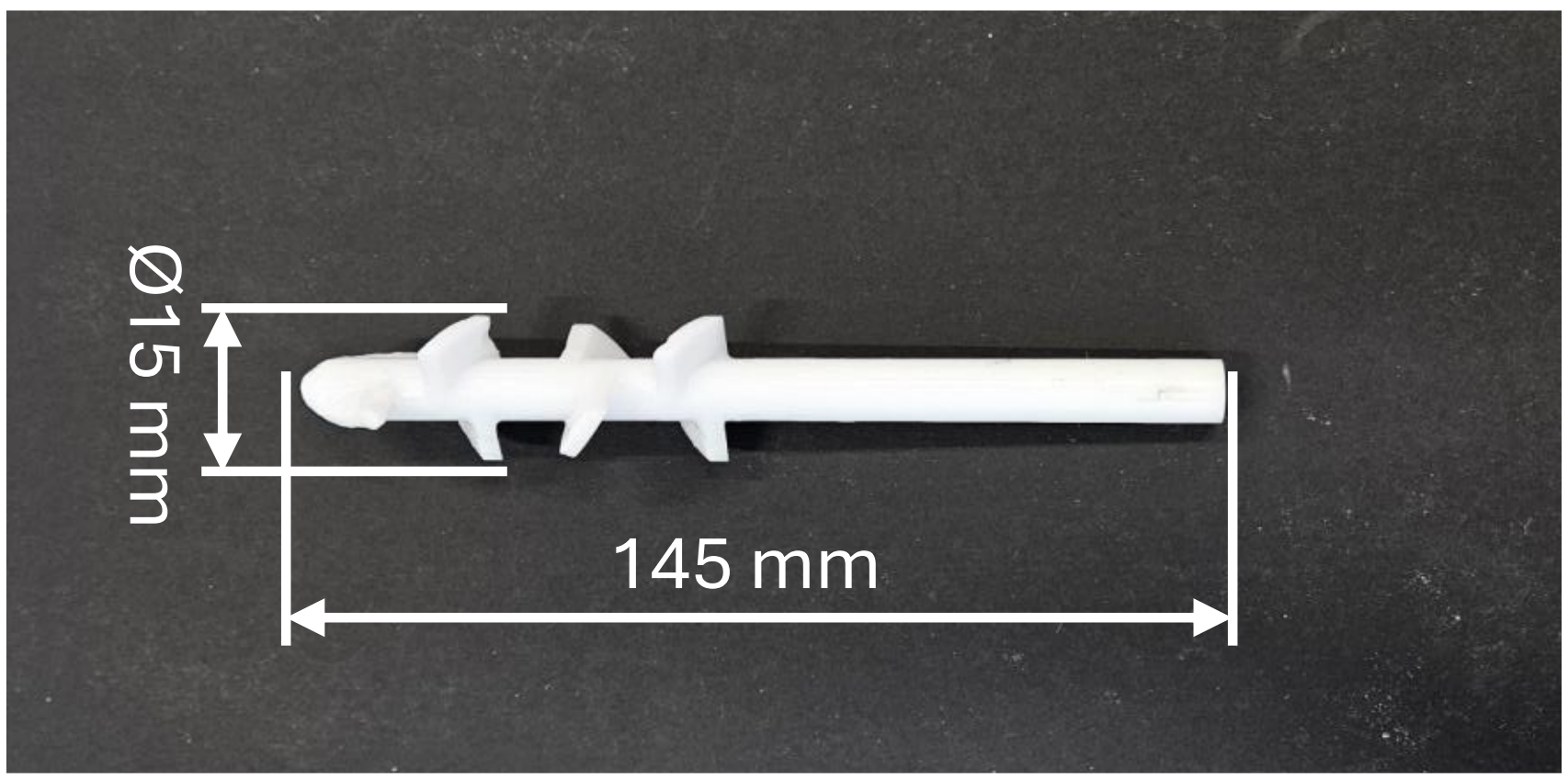

Figure S1. Custom-designed high-speed stirrer (maximum 2000 rpm) compatible with 50 mL conical centrifuge tubes. The stirrer was 3D printed using an ANYCUBIC resin printer and ABS-like resin.

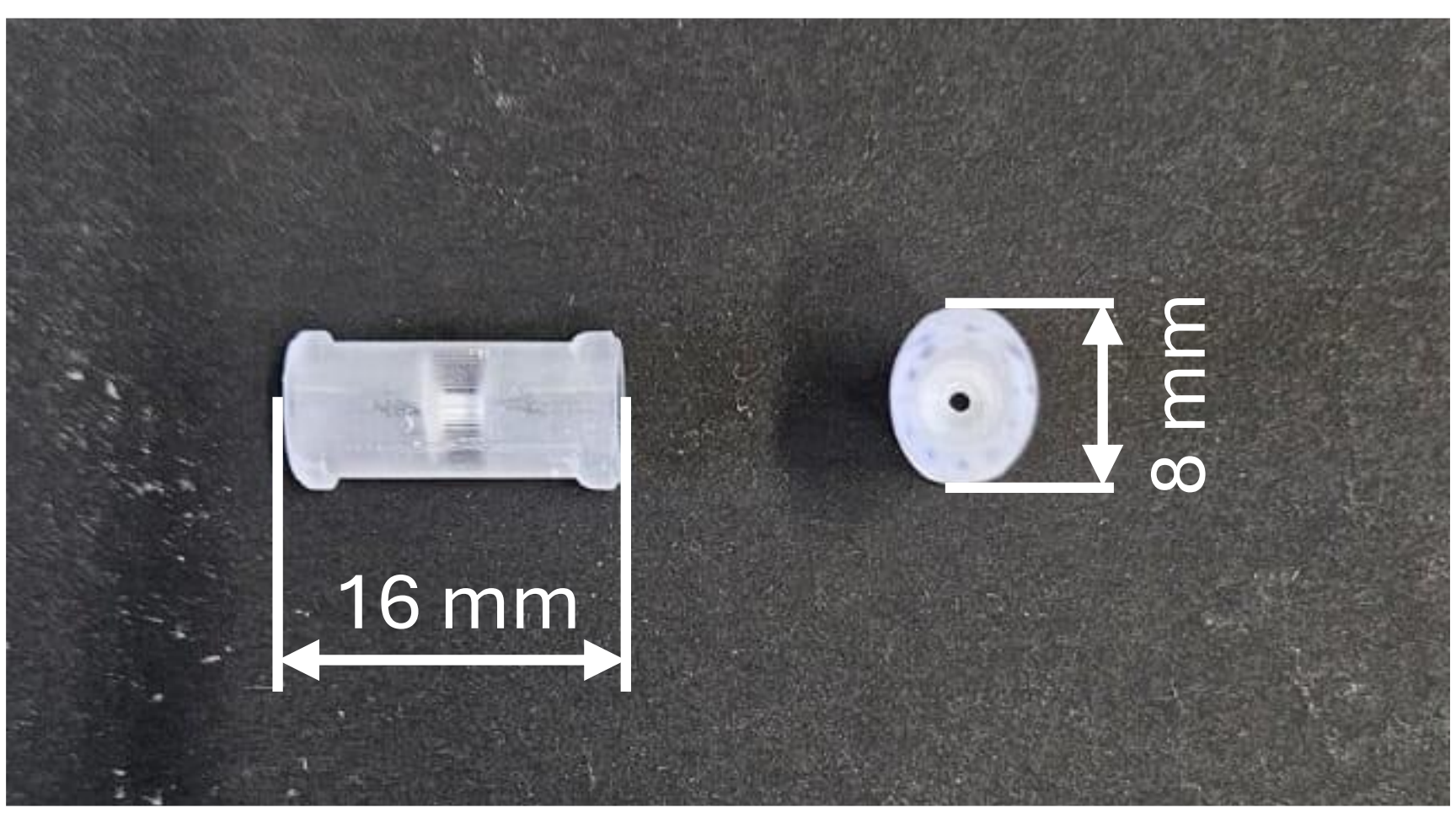

Figure S2. Custom-designed syringe–cartridge connector, used for the hygienic and efficient transfer of hydrogel ink from a syringe to an extrusion cartridge. The connector was 3D printed using an ANYCUBIC High Clear Resin.

2. Rheology

Rheological characterization of the hydrogel inks was performed using an Anton Paar Modular Compact Rheometer MCR 502. All measurements were conducted at 25°C using a parallel plate geometry.

Viscosity curves were recorded under steady shear conditions with a shear rate ramp from 0.1 to 100 s^{-1} over 30 seconds. Twenty-one data points were collected during this sweep.

Amplitude sweep tests were performed in oscillatory mode at a constant angular frequency of 10 rad/s. The shear strain amplitude was increased logarithmically from 0.01% to 100% over 25 data points. Measurements were taken to evaluate the linear viscoelastic regime and yielding behavior.

Temperature control and sample equilibration ensured consistent thermal conditions throughout the experiments.

3. Design, support generation, and slicing workflow

The digital pipeline used to generate printer-ready toolpaths for hydrogel direct ink writing (DIW) is presented, encompassing model import (STL or 3MF), support generation, slicing, and G-Code transfer to the GeSiM BioScaffolder. Autodesk Fusion 360 (Build v.2602.1.14) was used for CAD modeling, and the GeSiM BioScaffolder's integrated slicer (Version: 1.17.2.4575) was used for slicing and automatic support generation. STL files were used for single-part geometries (Tubes and test lines) and for community models obtained from public repositories. For multi-material jobs that included both structural and support components, 3MF was used to preserve part separation so that the slicer could correctly distinguish materials, which were then assigned to the respective cartridges.

Models were either designed in Fusion 360 and exported as STL or 3MF, or imported directly as STL when sourced from public repositories. After import into the GeSiM slicer, the anatomical aorta model was first examined prior to support generation to identify overhangs and internal cavities likely to require support (Figure S3a). Automatic support generation was then applied within the GeSiM slicer, producing supports beneath overhangs and within internal features, which are shown in the post-processing view of the same model (Figure S3b). For the 3D Benchy, we

first added targeted features in Fusion 360 to increase and localize support coverage in critical regions. The Benchy prior to support generation, with CAD-added features (rectangular blocks seen above Benchy), is shown in Figure S3c. Following import of this modified model, automatic support generation in the GeSiM slicer was performed, and the resulting support placement is shown in Figure S3d. The specific support auto-generation parameters used in this work (infill distance, outline distance, infill offset, clearance distance to the model, pressure settings, printing speed, layer height, and material assignment) are documented in a settings screenshot (Figure S3e), and these were applied consistently across the presented examples.

Slicing and parameterization were performed in the GeSiM integrated slicer. We set DIW-relevant parameters, including layer height, line width, extrusion pressure, print speed, infill distance, and configured support parameters as indicated in the settings panel (Figure S3e). Toolpath previews were used to verify support coverage and the correct sequencing of multi-material operations, as evidenced by the pre-support (Figure S3a, Figure S3c) and post-support (Figure S3b, Figure S3d) views. Once validated, printer-ready toolpaths (G-code) were uploaded to the BioScaffolder via USB connection. Prior to printing, we performed routine checks such as origin setting, nozzle offsets, layer height verification, and a dry-run preview where applicable.

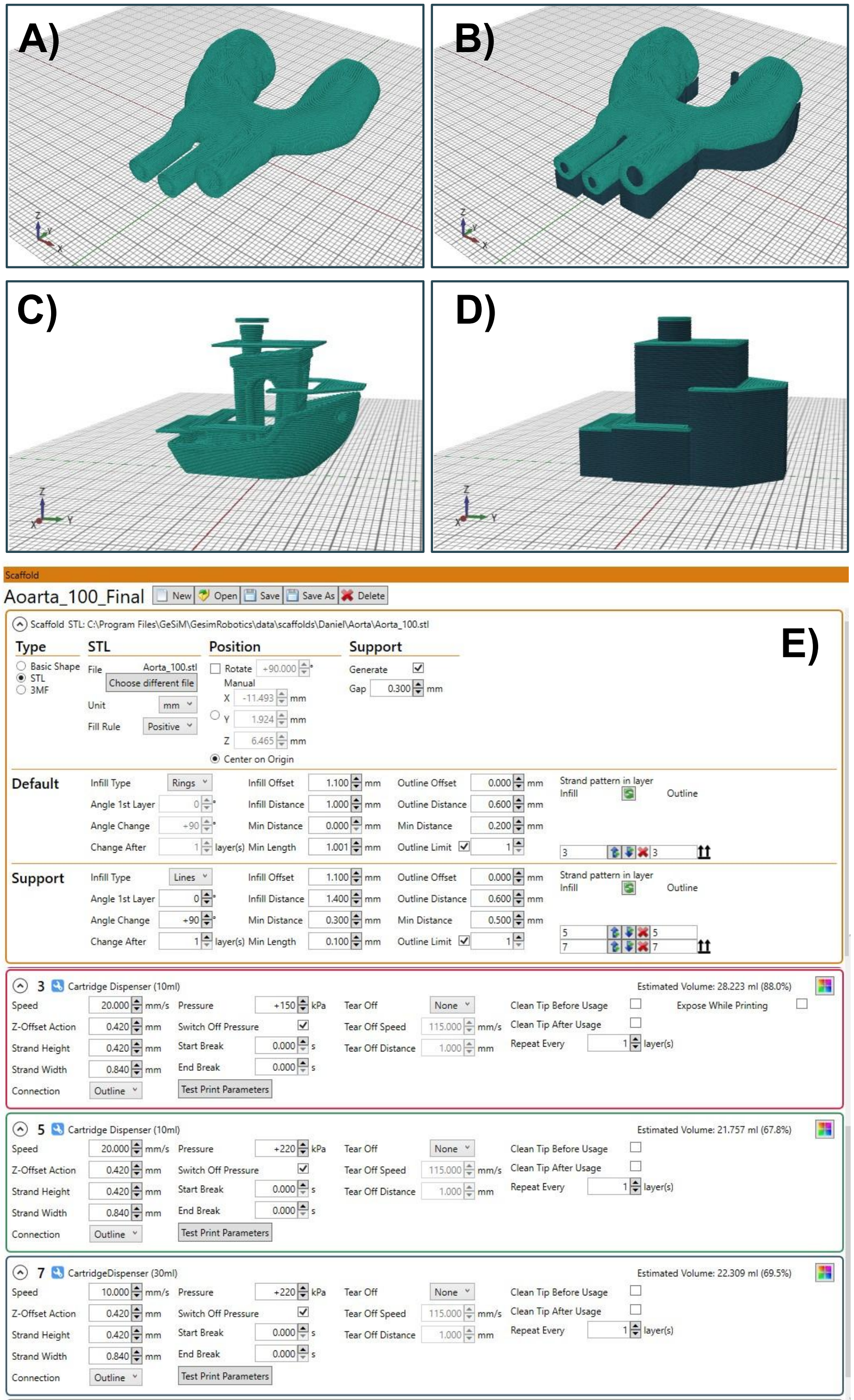

Figure S3. Digital workflow and automatic support generation: **A)** Anatomical aorta model after import (3MF), prior to support generation. **B)** Same aorta model following GeSiM automatic support generation, showing supports beneath overhangs and within internal features. **C)** 3D Benchy model prior to support generation; targeted features were added in Fusion 360 to increase and localize support coverage. **D)** Benchy after automatic support generation in the GeSiM slicer. **E)** Screenshot of the slicer and support auto-generation settings used in this work: infill distance, outline

distance, infill offset, clearance distance to the model, pressure settings, printing speed, layer height, and material assignment (structural vs. support). For multi-material printing, models and supports were exported as 3MF to preserve part separation and ensure correct material mapping during slicing.

4. 3D printing

Video S1. Timelapse of 3D printing of a hydrogel aorta structure using the GeSiM Robotics BioScaffolder 3.2. The red/pink ink corresponds to the structural nanofibrillated cellulose/sodium alginate (NFC/ALG) hydrogel ink, while the white ink represents the sacrificial NFC/hydroxyethyl cellulose/$CaCl_2$ (NFC/HEC/$CaCl_2$) support ink. The video demonstrates layer deposition and support integration. Real printing time: 4 h 38 min.

Video S2. Alternative timelapse view of the 3D printing of the hydrogel aorta structure using the GeSiM Robotics BioScaffolder 3.2. As in Video S1, the red/pink ink corresponds to the structural NFC/ALG hydrogel and the white ink to the sacrificial NFC/HEC/$CaCl_2$ support ink. This video presents the process from a different camera angle and at a different timelapse speed to highlight the overall printing dynamics and layer deposition.

5. References

(1) Lackner, F.; Knechtl, I.; Novak, M.; Nagaraj, C.; Dobaj Štiglic, A.; Kargl, R.; Olschewski, A.; Stana Kleinschek, K.; Mohan, T. 3D-Printed Anisotropic Nanofiber Composites with Gradual Mechanical Properties. Adv. Mater. Technol. 2023, 8 (10). https://doi.org/10.1002/admt.202201708.
